# Public health round-up

**DOI:** 10.2471/BLT.24.010824

**Published:** 2024-08-01

**Children’s hospital attacked in Ukraine**

A devastated ward in Ohmatdyt Hospital, the largest children's hospital in Kyiv, Ukraine. The hospital was partially destroyed on 8 July 2024 following a series of military strikes from the air.

**Figure:**
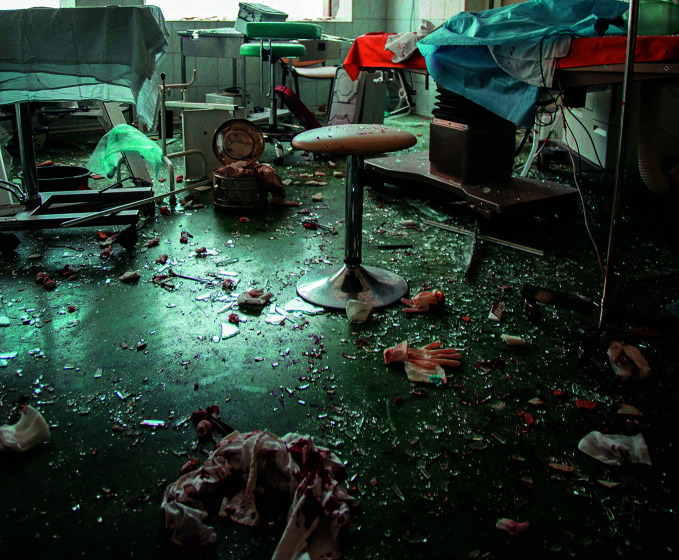
WHO

## Attack on children’s hospital in Ukraine

The Ohmatdyt National Children’s Hospital in Kyiv was severely damaged following a series of aerial strikes that took place on 8 July. More than 600 children were in the hospital when it was struck. Two people were killed and 50 injured, including 8 children. Sixty-eight children remained under treatment in the remaining buildings, while 94 children were transported to other medical facilities in Kyiv.

In an 11 July media briefing, World Health Organization (WHO) Director-General Tedros Adhanom Ghebreyesus reported that a WHO team arrived at the hospital immediately after the attack and that WHO biomedical engineers had assessed damage to medical equipment to determine what was needed to ensure continuity of care. WHO also provided medical supplies to hospitals that received patients from Ohmatdyt.

Since the beginning of the war, WHO has verified 1885 attacks on health care in the country, linked to 157 deaths and 435 injuries among health workers and patients.

https://bit.ly/3S3IrQl

## WHO response in Sudan

WHO’s African and Eastern Mediterranean Regional Offices staff met in Chad to assess the health needs of refugees fleeing the ongoing conflict and related humanitarian crisis in neighbouring Sudan. According to a 10 July media release, the mission aims to optimize WHO operations in Chad and Sudan with a view to providing essential medical care and enhancing cross-border humanitarian assistance.

In the last six months, the number of people experiencing high levels of acute food insecurity as a result of the crisis has surged by 45%, from 17.7 million to 25.6 million.

https://bit.ly/3VWVswt

## Immunization coverage stalls

Global childhood immunization coverage stalled in 2023, leaving 2.7 million additional children unvaccinated or under-vaccinated compared to pre-pandemic levels in 2019.

These estimates of national immunization coverage were published by WHO and UNICEF on 15 July. The estimates represent the most comprehensive dataset on immunization trends for vaccinations against 14 diseases and underscore the need for ongoing catch-up, recovery and system-strengthening efforts.

The number of children who received three doses of the vaccine against diphtheria, tetanus and pertussis (DTP) in 2023 stalled at 108 million. The number of children who did not receive a single dose of DTP vaccine increased from 13.9 million in 2022 to 14.5 million in 2023.

https://bit.ly/4bIxURP

## Physical inactivity rises

Nearly one third (31%) of adults worldwide, approximately 1.8 billion people, did not meet the recommended levels of physical activity in 2022. This is one of the key findings of an assessment published in *Lancet Global Health *on 25 June.

The findings point to a worrying trend of physical inactivity among adults, which increased by about 5% between 2010 and 2022. If the trend continues, levels of inactivity are projected to further rise to 35% by 2030.

On 26 June, WHO published a summary of the main findings, identifying the countries assessed to be on track to achieve the 2030 target for reducing physical inactivity and setting out six key policy actions.

https://bit.ly/3W3XrPs

https://bit.ly/3Lltp4M

## Falsified semaglutide

WHO issued a medical product alert regarding falsified semaglutide, a medication that mimics the action of a hormone which helps regulate blood sugar levels. The drug is used in the treatment of type 2 diabetes, but is also used off-label as an appetite suppressant to treat obesity.

The alert covered three falsified batches of the Ozempic brand which were detected in Brazil and the United Kingdom of Great Britain and Northern Ireland in October 2023, and the United States of America in December 2023. Since 2022, WHO's Global Surveillance and Monitoring System (GSMS) has noted an increase in reports of falsified semaglutide products across all regions.

https://bit.ly/466sleF

## Medical devices platform

WHO launched the first global open access platform designed to support governments, regulators and users in their decision-making on the selection, procurement and use of medical devices for diagnostics, testing and treatment of diseases and health conditions.

Launched on 8 July, the Medical Devices Information System (MeDevIS) includes 2301 types of medical devices.

“The number of medical technologies used in health care is growing, as is their complexity, which can make it challenging for health care practitioners and patients to navigate,” said Yukiko Nakatani, WHO Assistant Director-General for Access to Medicines and Health Products. “We aim to provide a one-stop shop of international information, which can be invaluable for those making decisions on life-saving medical technologies”.

https://bit.ly/3W75s6n

## Chad eliminates sleeping sickness

WHO congratulated Chad for having eliminated the gambiense form of human African trypanosomiasis, also known as sleeping sickness,* *as a public health problem.

The disease is the first neglected tropical disease to be eliminated in the country, and Chad is the first country to be acknowledged for eliminating a neglected tropical disease in 2024, becoming the 51^st^ country to have achieved such target globally, and marking the first step beyond the midpoint to the global threshold of 100 countries set for 2030.

https://bit.ly/3S6T2dE

## Substance use disorders

Some 2.6 million deaths per year are attributable to alcohol consumption, accounting for an estimated 4.7% of total deaths, while an estimated 0.6 million are due to psychoactive drug use. Males accounted for an estimated 2.0 million (83%) of deaths attributed to alcohol consumption and an estimated 0.4 million (66%) attributed to drug use.

This burden is according to WHO’s *Global status report on alcohol and health and treatment of substance use disorders *which was published on 25 June and provides a comprehensive update based on 2019 data.

The report shows an estimated 400 million people live with alcohol use disorders globally, of which some 209 million people are living with alcohol dependence.

https://bit.ly/3LmCvOM

## WHO prequalifies first self-test for hepatitis C virus

WHO prequalified the first hepatitis C virus (HCV) self-test. The test will provide a critical boost to expanding access to testing and diagnosis, accelerating global efforts to eliminate hepatitis C.

The product is an extension of the OraQuick® HCV rapid antibody test which was initially prequalified by WHO in 2017 for professional use.

WHO recommended HCV self-testing in 2021 to complement existing HCV testing services.

https://bit.ly/45ZD4aQ

**Cover photo**

A woman carrying what is left of her belongings, following a flash flood in Mai Mahiu, Kenya.

**Figure:**
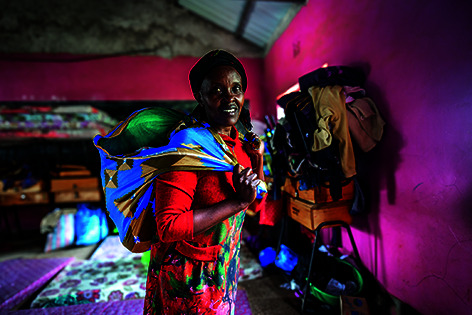
WHO/Genna Print

## Biosecurity guidance

WHO issued updated guidance on 21 June for national authorities and biomedical laboratories on the management of biological risks. The updated guidance includes recommendations regarding the strengthening of cybersecurity measures and the handling of confidential information such as patient records.

It also presents approaches to reducing risks from new technologies, including those related to genetic modification and manipulation of pathogens, and artificial intelligence (AI). There is also advice on keeping laboratories safe and secure during emergencies.

https://bit.ly/3zEEY4H

## Tobacco cessation guidance

WHO issued guidance on a comprehensive set of tobacco cessation interventions, including behavioural support delivered by health-care providers, digital cessation interventions and pharmacological treatments in a first guideline on tobacco cessation.

Released on 2 July, the guidance is relevant for all adults seeking to quit various tobacco products, including cigarettes, waterpipes, smokeless tobacco products, cigars, roll-your-own tobacco and heated tobacco products.

https://bit.ly/3Y13CGS

**Looking ahead**

15–16 August. The 10th International Conference on Public Health. Bangkok, Thailand. https://bit.ly/3y7QODL

16–17 August. Machine Learning for Healthcare. University of Toronto, Canada. https://www.mlforhc.org/

2–4 September. 15th World Conference on Injury Prevention and Safety Promotion. New Delhi, India. https://bit.ly/3Wnh06W

